# Ultrasound-guided needle positioning for nodal dynamic contrast-enhanced MR lymphangiography

**DOI:** 10.1038/s41598-022-07359-1

**Published:** 2022-03-07

**Authors:** J. Wagenpfeil, P. A. Kupczyk, A. Henkel, S. Geiger, T. Köster, J. A. Luetkens, H. H. Schild, U. I. Attenberger, C. C. Pieper

**Affiliations:** Department of Diagnostic and Interventional Radiology, University Hospital Bonn, Venusberg-Campus 1, 53127 Bonn, Germany

**Keywords:** Diseases, Medical research

## Abstract

The aim of the study was to assess injection needle positioning for contrast-enhanced MR-lymphangiography (MRL) by ultrasound-guided injection of saline-solution. 80 patients (33 male, mean age 43.1 years) were referred for MRL. The injection needle position was assessed by injection of saline-solution. Consecutive lymph node distension was observed on sonography followed by MRL. Transpedal MRL was performed when no inguinal lymph nodes could be identified. The inguinal lymph node detection rate was recorded. MR-lymphangiograms were assessed regarding primary (i.e. enhancement of draining lymph vessels) and secondary technical success (i.e. lymph vessel enhancement after repositioning of the needle). MRL was considered as clinically successful if enhancement of the central lymphatic system and/or a lymphatic pathologies were observed. For a total of 92 MRLs 177 groins were evaluated sonographically. In 171/177 groins (96.6%) lymph nodes were identified. After needle placement lymph node distension was observed in 171/171 cases (100%) on saline injection. MR-contrast injection demonstrated enhancement of draining lymph vessels in 163/171 cases (95.3%). In 6/171 cases (3.5%) in-bore needle retraction lead to lymphatic enhancement. In one patient [2/171 nodes (1.1%)] no lymphatic enhancement was seen despite repeated needle repositioning. Overall contrast application was technically successful in 169/171 cases (98.8%). In the 6 groins in which no nodes were identifiable, transpedal MRL was successful. So overall 91/92 MRLs (98.9%) were clinically successful. No complications were recorded. Confirmation of the needle position for nodal MRL by sonographically controlled saline injection is a reliable technique with a high success rate of MRL.

## Introduction

For decades lymphatic imaging has been limited to transpedal x-ray lymphangiography and lymphoscintigraphy. Introduction of nodal x-ray lymphangiography with ultrasound-guided lymph node access has led to a broader adoption of lymphatic imaging and interventions. However, x-ray lymphangiography in general is associated with possible complications of the oil-based contrast-agent^1,2^. For diagnostic purposes MR-lymphangiography (MRL) offers a less invasive alternative and can be performed with either nodal or interstitial transpedal contrast-application^3,4^. By identifying lymphatic anatomy MRL is a useful tool especially in pre-treatment work-up of patients with central lymphatic pathologies (e.g. chylothorax)^4,5^. Nodal dynamic contrast-enhanced MRL (DCE-MRL) can show the dynamics of lymph flow with both high temporal and spatial resolution, and is therefore particularly useful in lymph flow disorders (e.g. plastic bronchitis)^6^. The most important factor for a successful DCE-MRL examination is a stable needle position in the punctured lymph node^1,4^. As repeated needle repositioning is usually time consuming because of the necessary transfers into and out of the MR-scanner, it is important to validate the needle positions before the actual MR-examination. Different techniques to validate the needle position within a lymph node have been described. However, these techniques either require a dedicated combined MR-angiography suite (so called X-MR suite) or the additional off-label application of ultrasound contrast agent^2,7,8^. The aim of the present study was therefore to evaluate needle position validation by sonography-guided injection of physiological saline instead of contrast-agent immediately before DCE-MRL.

## Materials and methods

In this retrospective study consecutive patients with clinically suspected lymphatic pathologies were included between 06/2018 and 09/2019. In all patients ultrasound-guided needle positioning was performed for transnodal MR-lymphangiography as part of our standard clinical work-up. Patients were informed about the procedure in detail especially the off-label use of the MR contrast agent for lymphangiography and provided written informed consent for the clinical MRL examination. The institutional review board of the Medical Faculty of the Rheinische Friedrich-Wilhelms University Bonn approved data analysis and waived additional informed patient consent for retrospective data analysis.

### Patient cohort

We reviewed 80 consecutive patients (47 female, 33 male; mean age 43.1 years, range 0.5 to 88.6 years) with clinically suspected lymphatic pathologies (see Table 1 for details). 72/80 patients underwent one, 6 patients two, one patient three and one patient five MRLs for a total of 92 MRL examinations, which are the basis of this study. Of the included patients 11 were children < 10 years and 5 were infants < 2 years.

**Table 1:** Patient characteristics.

Variable	Value (%)
Number	80
**Sex**	
Male	33 (41.3)
Female	47 (58.8)
Mean age (years ± SD)	43.1 ± 26.2
**Clinical indication for MR-lymphangiography**	
Chylothorax	28 (35)
Chylous ascites	13 (16.3)
Combined chylothorax/chylous ascites	6 (7.5)
Pelvic lymphatic fistula	6 (7.5)
Complications from congenital heart disease	11 (13.8)
Lymphatic malformations	4 (5)
Localized lymphedema (e.g. genital)	12 (15)

### MR lymphangiography

MRL was performed on a 1.5-T system (Ingenia; Philips Healthcare, Best, The Netherlands) with the patients on a detachable mobile MR table in supine position. In young children the examination took place under general anesthesia if necessary.

**Figure 1:**
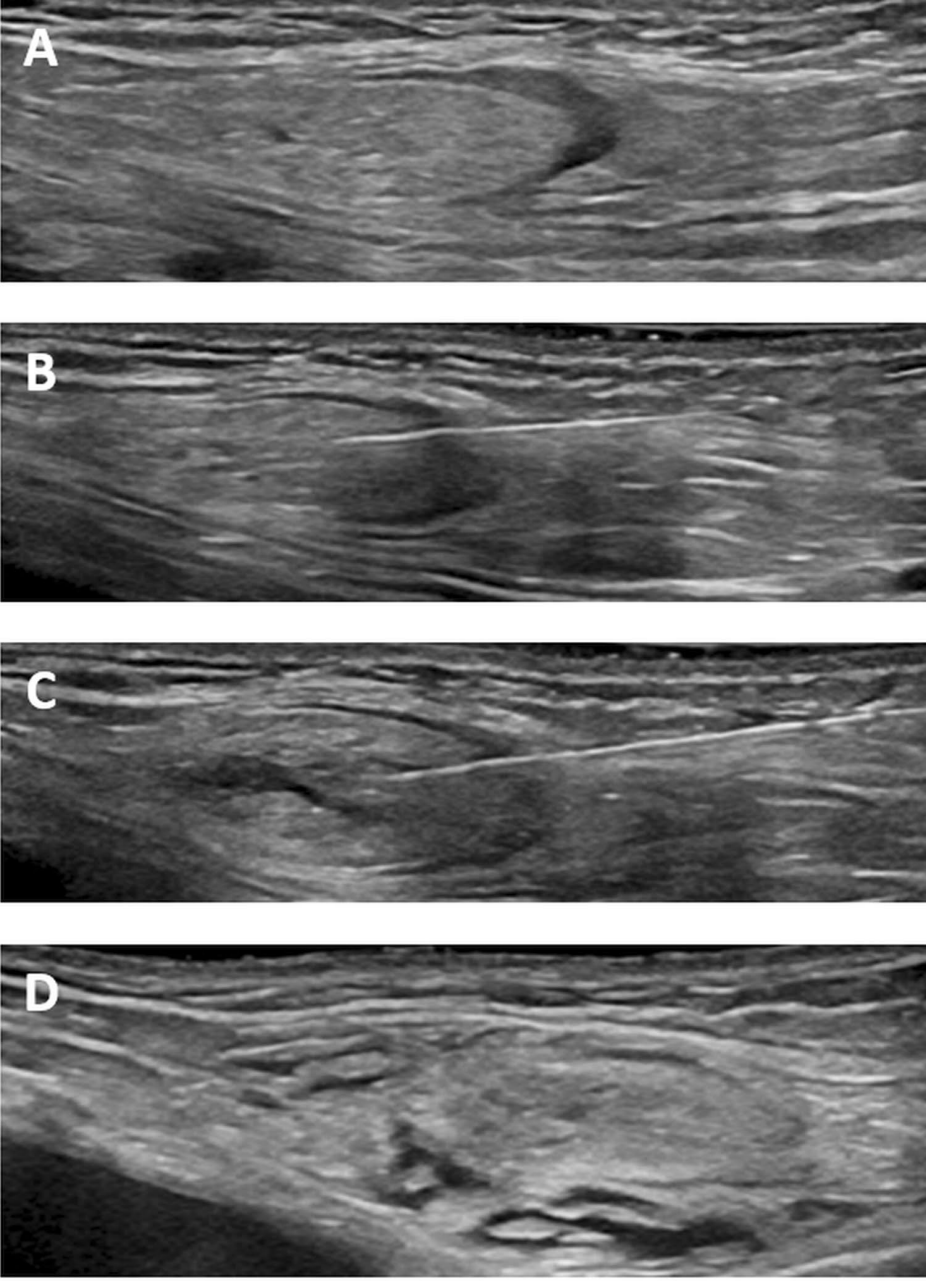
Ultrasound images demonstrating (**A**) a groin lymph node before puncture, (**B**) after puncture with a 25-gauge puncture needle with its tip in the transitional zone, (**C**) lymph node distension after injection of a small amount of saline solution and (**D**) saline extravasation without lymph node distension (different patient than (**A–C**)).

After non-contrast imaging, the patients were transferred out of the scanner room and the groins were prepared for sterile puncture. Under real-time ultrasound guidance inguinal lymph nodes (either bilateral or unilateral, depending in the indication for examination) were identified using a linear 18 MHz probe (LOGIQ Vivid E90, GE Healthcare) (Fig. 1a).

All ultrasound-guided lymph node punctures were performed by the same interventional radiologist (C.C.P. with 10 years of interventional experience). A 25-gauge spinal needle (BD Medical, USA) was advanced percutaneously towards a lymph node in a shallow angle; this ensured a relatively long subcutaneous needle tract improving needle stability. The needle tip was positioned in the transitional zone between the cortex and hilum of the lymph node (Fig. 1b).

To minimize movement the needle was already prepared with a short piece of extension tubing connected to a three-way stopcock with one syringe containing diluted 1.0 mmol/mL gadobutrol (Gadovist, Bayer Healthcare, Germany; diluted 1:2 with physiological saline) as described previously^5^, and one syringe filled with saline.

To check the needle position within the lymph node, 1 to 2 ml of physiological saline solution were carefully injected under ultrasound observation. With correct needle placement lymph node distension (increase in lymph node size) was observed (Fig. 1c). If no distension was seen or extravasation was detected (Fig. 1d), the needle was repositioned immediately. At the discretion of the interventionalist this consisted of either moving the needle tip 1–2 mm or repeat puncture with needle placement into a different part of the lymph node or a different lymph node.

With satisfactory needle position patients were transferred back into the MR scanner. MRL was performed with continuous and slow application (approx. 1 ml per minute) of the diluted contrast agent with repetitive acquisition of a coronal T1-weighted sequence at time intervals of 45 s (TR 5.2 ms, TE 1.8 ms and 4 ms, flip angle 20°, field of view: 430 mm, matrix: 480 × 480 mm, acquisition time per stack 40 s).

If lymph vessel enhancement did not occur or primarily venous drainage of the contrast agent was seen on initial MR-images, repositioning of the respective needle was carried out. Therefore the needle tip was retracted 1–2 mm with continuation of the MR examination (in-bore needle retraction). If lymphatic enhancement was still inadequate, the patient was transferred back to sonography for guided needle repositioning.

In patients without accessible lymph nodes on ultrasound imaging, transpedal interstitial MRL was performed as described elsewhere^5^.

### Data acquisition

Clinical (patient age, gender, clinical indication for MRL) and procedural (number of MRLs per patient, MRL technique [bilateral, unilateral; nodal, transpedal]) data were collected from the electronic patient files.

The rate of groin lymph node detection was recorded from ultrasound examination results. The short diameter of the punctured lymph node was measured perpendicular to the long axis on the documented sonographic images prior to saline injection.

**Figure 2:**
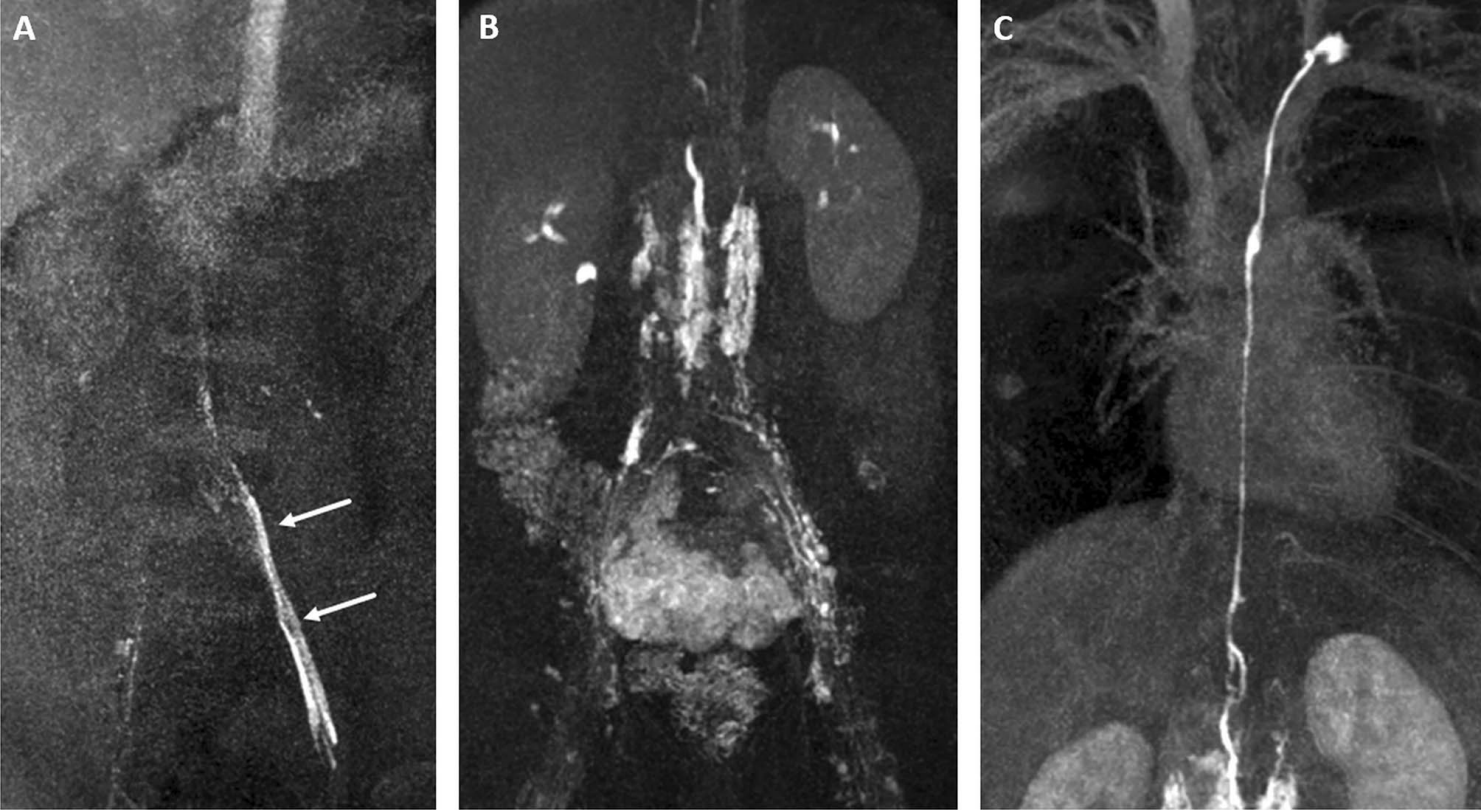
Maximum intensity projection (MIP) images of MR-lymphangiograms showing (**A**) marked venous enhancement on the left side with enhancement of the left iliac vein (arrows), making needle repositioning necessary (retraction by 1–2 mm). Initial lymphatic enhancement can be seen on the right side. After needle retraction adequate bilateral lymph flow into the lymphatic system with enhancement of (**B**) pelvic and retroperitoneal as well as (**C**) thoracic lymphatics can be observed.

The recorded MR-lymphangiograms were retrospectively reviewed by two radiologists in consensus (C.C.P. and J.W. with 10 and 5 years of experience, respectively) regarding:

- Enhancement of iliac, retroperitoneal and thoracic lymph vessels and nodes
- Venous enhancement originating from the injection sites (i.e. enhancement of iliac veins and inferior vena cava) as sign of venous drainage from the punctured lymph node; venous enhancement was graded as either minimal or marked venous run-off) (Fig. 2a).

The frequency and technique of necessary needle repositioning steps (in-bore needle retraction or sonographically guided repositioning) were recorded.

Peri-interventional complications of lymph node puncture and contrast medium injection were recorded if present.

### Definitions

Ultrasound-guided needle placement was considered successful when lymph node distension was observed upon saline injection.

When MR contrast medium injection revealed enhancement of the respective iliac lymph vessels after ultrasound-guided needle placement, this was defined as primary technical success. This included cases with minimal venous enhancement originating from the injection site. Secondary technical success was defined as successful enhancement of the respective iliac lymph vessels after at least one needle repositioning (either in-bore needle retraction or sonographically guided repositioning).

The MRL examination was considered clinically successful when enhancement of the central lymphatic system (retroperitoneal and/or the thoracic duct) and/or a lymphatic pathology preventing central lymphatic enhancement was seen.

### Statistical analysis

Statistical analyses were performed using SPSS, version 23.0 (IBM, Armonk, NY). Descriptive statistics were performed for patient characteristics and imaging findings given as mean ± standard deviation for continuous variables, median and range for skewed continuous variables or count for categorical variables. Interrelations between primary technical success and shortest diameter of the punctured lymph node was assessed by Wilcoxon U test.

### Ethical approval and informed consent

The presented study was approved by the institutional review board of the Medical Faculty of the Rheinische Friedrich-Wilhelms University of Bonn and hence all methods were performed in compliance with the ethical standards set in the 1964 Declaration of Helsinki as well as its later amendments. Written informed consent for retrospective data analysis was waived.

## Results

85/92 MRLs were performed with a bilateral, 7/92 with a unilateral access (6 pelvic lymphatic fistulas, 1 lymphatic malformation), so that overall 177 groins were evaluated by ultrasound for possible lymph node puncture. Results of needle placements and MRL examinations are summarized in Table 2.

**Table 2:** Results of ultrasound guided needle placement and MR contrast medium injection.

	Number of groins (%)	Number of MRLs (%)
Total	177	92
**Ultrasound needle positioning results**		
Lymph nodes detectable	171/177 (96.6%)	89/92 (96.7%)
Attempted nodal needle placements	171	89
Lymph node distension	171/171 (100%)	89/171 (100%)
**MRL results**		
Nodal CM-injection	171	89
Technical success	169/171 (98.8%)	88 (98.8%)
Primary success	163/171 (95.3%)	84 (94.4%)
Secondary success	6/171 (3.5%)	4 (4.5%)
No technical success	2/171 (1.2%)	1 (1.1%)
Transpedal CM-injection	6	3
Technical success	6/6 (100%)	3/3 (100%)
Overall success	175/177 (98.9%)	91/92 (98.9%)

Lymph nodes were identified in 171/177 (96.6%) groins (32/32 [100%] in pediatric patients) and ultrasound-guided needle placement was attempted. After needle placement lymph node distension was detected by ultrasound in all (171/171) cases upon saline injection indicating an adequate needle position. The mean transverse diameter of punctured lymph nodes was 6.7 ± 2.9 mm (range 1–15 mm).

After transfer into the MR unit these 171 lymph nodes were injected with MR contrast medium according to our protocol. In 163/171 (95.3%) lymph nodes MR revealed free flow into draining lymph vessels leading to adequate enhancement of iliac, retroperitoneal and thoracic lymphatics (primary technical success). In 4 of these 163 cases slight venous enhancement was observed in addition to lymph vessel delineation, which required no further measures.

In 6/171 (3.5%) lymph nodes marked venous enhancement was detected (Fig. 2a), which necessitated in bore needle repositioning (retraction by 1–2 mm). In all cases adequate lymphatic enhancement was obtained by this correction (secondary technical success) (Fig. 2b,c).

In 2/171 lymph nodes (1.1%) in the same patient only venous enhancement, but no lymphatic enhancement was achieved despite adequate lymph node distension on saline injection. Repeated needle repositioning in the same as well as different lymph nodes did not result in enhancement of lymph vessels.

Overall 169/171 (98.8%) contrast injections were technically successful including all injections in pediatric patients.

In 6/177 (3.4%) groins in three patients no groin lymph nodes could be detected on sonography due to an extensive inguinal lymphedema (n = 1), radiogenic changes (n = 1) and inguinal lymph node extirpation (n = 1). In these cases transpedal contrast medium application was performed according to our protocol. All of these three transpedal MRLs were successful with enhancement of iliac, retroperitoneal and/or thoracic lymph vessels.

Overall 91/92 MRL-examinations (98.9%) were clinically successful depicting the central lymphatic system and/or a lymphatic pathology (87/88 nodal, 3/3 transpedal).

Statistical analysis did not show a significant difference in lymph node size between the groups with and without technical success of contrast injection (6.6 ± 2.9 mm vs. 7.3 ± 3.5 mm, p = 0.64).

There were no complications from lymph node puncture, saline or contrast injection within an observational period of at least 1 week.

## Discussion

Techniques for imaging of lymphatic vessels and lymphatic interventions have evolved rapidly in recent years. Nodal dynamic contrast-enhanced MR lymphangiography is a novel, minimally invasive method to depict the central lymphatic system enabling the evaluation of various lymphatic diseases, such as chylothorax, chylous ascites or plastic bronchitis^5,6^. By identifying lymphatic anatomy and—when performed as a dynamic study—providing functional information about lymph flow, DCE-MRL is also useful for planning interventional procedures. When employing this technique, a correct and stable needle position in a lymph node is of utmost importance to ensure enhancement of the lymphatic system.

The first step of this procedure, puncture of a groin lymph node under ultrasound guidance, has a very high success rate. In our cohort all attempted lymph node punctures were successful. Correct needle position can be demonstrated in various ways. When fluoroscopy is used, this requires the injection of iodinated contrast agent^2^, and a fluoroscopy unit in proximity to the MR scanner or an X-MR suite, which however exists only in a very few centers. Needle position may also be checked by ultrasound and (off label) injection of ultrasound contrast agent visualizing its lymphatic run-off^6^. While obviating the need for ionizing radiation and iodinated contrast agent, this technique may be associated with (rare) side effects of ultrasound contrast agent and its increased costs^9^.

In our study we analyzed, if ultrasound guided injection of physiological saline instead of ultrasound contrast agent may also reliably allow for assessment of correct needle placement. The main result of the present study was that lymph node distension is a reliable sign of correct needle positioning. In 163/171 cases with lymph node distention adequate and uncomplicated lymphatic run-off was shown by MR. In additional 6/171 cases only minimal (in-bore) needle retraction was necessary to achieve adequate lymphatic enhancement on MRI.

In comparison, Nadolski and co-workers performed needle placement in groin lymph nodes in 28 patients using a comparable puncture technique as in the present study with the patients also on a detachable MR table. Ultrasound contrast agent was used to visualize efferent lymphatics as a sign of adequate needle position. After ultrasound contrast injection needle repositioning was necessary in 6/28 patients. In the subsequent MR contrast-injection was successful in all cases^6^.

In our cohort a varying degree of venous enhancement was observed in 10/171 punctures originating from the injection site, but necessitated needle repositioning in only 6/171 of cases. A possible explanation for venous enhancement may be lymphatico-venous shunts at the level of the lymph node as described by Kariya et al.^10^. However, marked venous enhancement without lymphatic enhancement may also result from a central needle position in the lymph node hilum. Therefore, positioning the needle tip in the corticomedullary junction of the lymph node has been recommended^1,4^. In contrast to saline injection, ultrasound contrast agent used for needle position validation may be advantageous in showing primary venous run-off already before MR contrast agent injection. This was demonstrated by Nadolski et al. in 1/28 cases with enhancement of the femoral vein on ultrasound contrast injection^7^. However, primary venous run-off was only visible in 6/171 cases in our cohort and could be resolved in all of these cases by simple needle retraction inside the scanner without the need for repeated ultrasound.

A stable needle position may be achieved by a long and shallow subcutaneous needle tract. Additional needle fixation has been reported^5,6^, but was not necessary in our experience with the patient on a detachable MR-table. We did not observe a secondary needle displacement when transferring the patients from ultrasound into the MR scanner.

Interestingly the lymph node size in our study did not show a significant association with technical success of MR contrast injection. MRL was unsuccessful in a patient with rather large lymph nodes (12–14 mm), but was successful in infants with lymph node diameters as low as 1 mm. The case with unsuccessful transnodal examination in our study remains unclear. As there was no demonstrable lymphatic drainage in this patient even after multiple needle repositioning with adequate lymph node distension on sonography, it is to be assumed that the cause of failure was the underlying lymphatic pathology rather than a technical failure.

Although lymph nodes can be identified on ultrasound in almost all patients^7^, nodal access may not be feasible in rare cases without detectable nodes. This was the case in three patients in our cohort and was due to extensive inguinal lymphedema, radiogenic changes or inguinal lymph node extirpation. In these cases transpedal MRL can be a viable alternative^3,5^ and was successful in all three patients. However, in comparison to nodal MRL, transpedal MRL usually yields inferior enhancement of retroperitoneal lymph vessels / thoracic duct due to the longer access path as well as limited temporal flow information^5^.

This study has several limitations. First, this was a retrospective study with inherent methodological limitation. Therefore, the number of initial sonographic needle positioning attempts to achieve lymph node distension prior to MRL as well as information about the duration of sonographically guided puncture were not available. Second, the study was performed at a single center with all lymph node punctures being performed by the same interventionalist which may impair generalizability of the results. Third, this study focused on needle positioning and not on the technique of transferring the patient securely without secondary needle dislocation. All MRLs were performed on a scanner with a detachable MR-table that allowed for minimal patient movement during transfer in and out of the scanner. Different techniques may be required if the examination table cannot be moved. Furthermore, lymph node diameter could only be measured on the documented sonographic images so that the lymph node axis may vary between cases.

In conclusion, confirming an adequate needle position inside lymph nodes for nodal MR lymphangiography by sonographically controlled saline injection is a straightforward and reliable technique leading to a high rate of successful MRL examinations without the need for special equipment or additional ultrasound contrast application.
